# Energy metabolism in *Trypanosoma cruzi*: the validated and putative bioenergetic and carbon sources

**DOI:** 10.1128/mbio.02215-24

**Published:** 2025-05-20

**Authors:** Mayke B. Alencar, Sabrina Marsiccobetre, Ana C. Mengarda, Maria Sol Ballari, Ariel M. Silber

**Affiliations:** 1 Laboratory of Biochemistry of Trypanosomatids (LaBTryps), Department of Parasitology, Institute of Biomedical Science II (ICB II), University of São Paulo, São Paulo, Brazil; Instituto Carlos Chagas, Curitiba, Brazil

**Keywords:** *Trypanosoma cruzi*, bioenergetics, Chagas disease, amino acid metabolism, carbohydrate metabolism, mitochondria, glycosome

## Abstract

*Trypanosoma cruzi,* along with *Trypanosoma brucei* and over 20 species of the genus *Leishmania,* constitutes a group of human pathogenic flagellated protists collectively called the “TriTryp,” posing among the best-studied protists. These organisms have complex life cycles and are transmitted by insects, which, along with vertebrates, serve as their natural hosts. Throughout their life cycles, these parasites encounter diverse environments with varying physical, chemical, biochemical, and biological characteristics that serve as stages for their evolutionary stories, culminating in different metabolic configurations and requirements. Here, we review the evidence for metabolic pathways that directly or indirectly participate in energy-transducing processes, discussing where appropriate the implications of the different metabolic networks in TriTryp.

## INTRODUCTION

**Fig 1:**
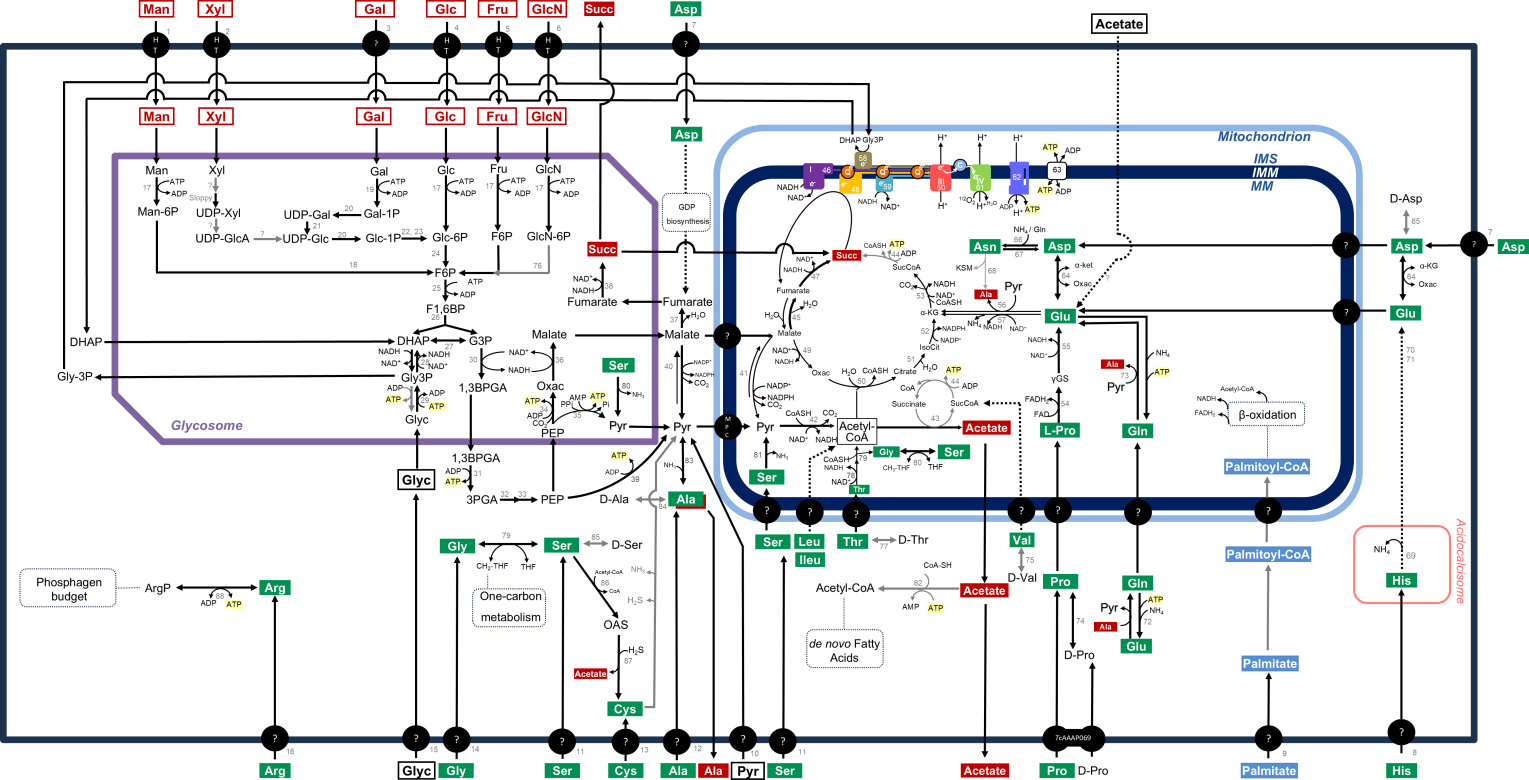
Network of carbon and energy metabolism of *Trypanosoma cruzi* epimastigotes. Amino acids are in white font on a green background, excreted products are in white font on a red background, carbohydrates are in red font on a white background, fatty acids are in white font on a blue background, and other carbon sources are in black font on a white background. Gray arrows represent putative steps, and dotted arrows indicate multiple enzymatic steps. IMS, mitochondrial intermembrane space; IMM, inner mitochondrial membrane; MM, mitochondrial matrix. Transport systems for carbon sources are 1, mannose; 2, xylose; 3, galactose; 4, glucose; 5, fructose; 6, glucosamine; 7, aspartate; 8, histidine; 9, palmitate; 10, pyruvate; 11, serine; 12, alanine; 13, cysteine; 14, glycine; 15, glycerol; and 16, arginine. Enzymes are 17, hexokinase; 18, mannose-6-phosphate isomerase; 19, galactokinase; 20, UDP-sugar pyrophosphorylase; 21, UDP-galactose 4-epimerase; 22, phosphoglucomutase; 23, phosphomannomutase; 24, glucose-6-phosphate isomerase; 25, phosphofructokinase; 26, aldolase; 27, triose-phosphate isomerase; 28, glycerol-3-phosphate dehydrogenase; 29, glycerol kinase; 30, glyceraldehyde-3-phosphate dehydrogenase; 31, phosphoglycerate kinase; 32, phosphoglycerate mutase; 33, enolase; 34, phosphoenolpyruvate carboxykinase; 35, pyruvate phosphate dikinase; 36, glycosomal malate dehydrogenase; 37, cytosolic fumarase; 38, glycosomal NADH-dependent fumarate reductase; 39, pyruvate kinase; 40, cytosolic malic enzyme; 41, mitochondrial malic enzyme; 42, pyruvate dehydrogenase complex; 43, acetate:succinate CoA-transferase; 44, succinyl-CoA synthetase; 45, mitochondrial fumarase; 46, NADH:ubiquinone oxidoreductase (respiratory chain complex I); 47, mitochondrial NADH-dependent fumarate reductase; 48, succinate dehydrogenase (respiratory chain complex II); 49, mitochondrial malate dehydrogenase; 50, citrate synthase; 51, aconitase; 52, isocitrate dehydrogenase; 53, α-ketoglutarate dehydrogenase; 54, proline dehydrogenase; 55, pyrroline-5-carboxylate dehydrogenase; 56, alanine aminotransferase; 57, glutamate dehydrogenase; 58, mitochondrial FAD-dependent glycerol-3-phosphate dehydrogenase; 59, rotenone-insensitive NADH dehydrogenase (NDH2); 60, ubiquinol-cytochrome C oxidoreductase (respiratory chain complex III); 61, cytochrome C oxidase (respiratory chain complex IV); 62, F_o_F_1_- ATP synthase; 63, ADP/ATP translocator; 64, aspartate aminotransferase; 65, aspartate racemase; 66, aspartate-ammonia ligase; 67, asparaginase; 68, asparagine-keto acid aminotransferase; 69, histidine ammonia lyase; 70, urocanate hydratase; 71, imidazolone propionase; 72, glutamine synthetase; 73, glutamine aminotransferase; 74, proline racemase; 75, valine racemase; 76, glucosamine-6-phosphate isomerase; 77, threonine racemase; 78, threonine dehydrogenase; 79, AKCT, 2-amino-3-ketobutyrate CoA-transferase; 80, glycine hydroxymethyltransferase; 81, serine dehydratase; 82, acetyl-CoA synthetase; 83, alanine aminotransferase; 84, alanine racemase; 85, serine racemase; 86, serine O-acetyltransferase; 87, cysteine synthase; 88, arginine kinase. Sloppy: PPase enzyme that utilizes diverse sugar 1-phosphates and UTP to form UDP-sugars. MPC, mitochondrial pyruvate carrier, TcAAAP069: proline permease.

Cellular metabolism is essential for all living organisms to transduce energy for maintenance and proliferation. Its configuration varies across species and is shaped by evolutionary adaptations to environmental challenges. Pathogenic trypanosomatids, including *Trypanosoma cruzi*, *Trypanosoma brucei,* and *Leishmania* spp. (collectively called TriTryps), cause Chagas disease, human african trypanosomiasis, and leishmaniasis, respectively. These parasites exhibit remarkable metabolic adaptations, enabling them to survive in diverse biochemical environments within their vertebrate and invertebrate hosts. For *T. cruzi*, these environments range from the glucose-rich vertebrate blood and the host-cell cytoplasm, where the parasite needs to compete for nutrients with the host-cell enzymatic machinery, to the severely nutrient-depleted insect midgut (1–4). Since diverging from the last eukaryote common ancestor (5, 6), TriTryps have evolved unique metabolic and structural innovations. A key example is the glycosome, a microbody-like organelle first described in *T. brucei* in 1977, which compartmentalizes enzymes for glycolysis and glycerol metabolism (7). This organelle is also found in most trypanosomatids, including *T. cruzi* (8), originated from peroxisomes (reviewed in reference 9) and later was shown to house enzymes from the pentose-phosphate pathway, glycerol-phosphate shuttle, lipid metabolism, purine salvage, and pyrimidine biosynthesis, among others (7, 10–13). Additionally, TriTryps possess a single and unique mitochondrion with DNA organized in a structure called the kinetoplast (14–16) and alternative energy-transduction mechanisms when compared to those of their hosts. This “peculiar” metabolic configuration allows them to thrive in the dynamic nutrient environments of their hosts. This review summarizes the available data on the uptake , biosynthesis, and catabolism of metabolites (Fig. 1), which fuel *T. cruzi*’s energy metabolism, aiming to provide a comprehensive “bioenergetics cartography” of this parasite.

### *Trypanosoma cruzi* life cycle

**Fig 2:**
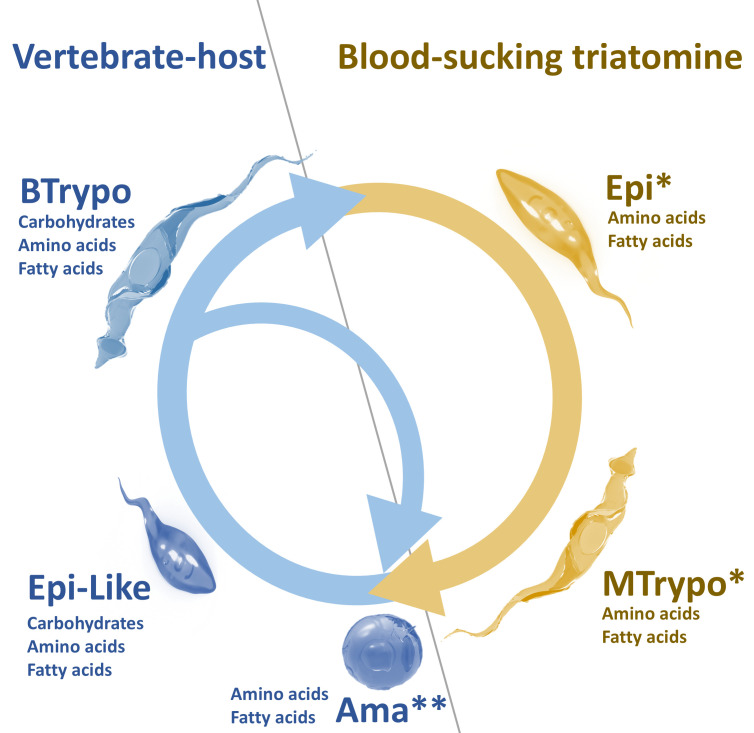
Life cycle of *Trypanosoma cruzi* and stage-specific type of carbon source utilization. The scheme depicts the parasite’s life cycle occurring between vertebrate and insect hosts, highlighting metabolic adaptations to environmental nutrient availability. In the vertebrate host, bloodstream trypomastigotes (BTrypo) extracellularly exploit carbohydrates (e.g., glucose) from the blood plasma, while intracellular amastigotes (Ama) scavenge fatty acids and amino acids from the host cytoplasm. In the triatomine insect vector, ingested BTrypo differentiates into replicative epimastigotes (Epi) in the midgut, utilizing amino acids and fatty acids. These transition to non-replicative metacyclic trypomastigotes (MTrypo) in the hindgut, which rely on stored fatty acids and amino acids to survive nutrient scarcity. MTrypo are transmitted to new vertebrate hosts via triatomine fecal contamination, invading host cells to reinitiate the cycle. Arrows indicate life cycle progression on the invertebrate (yellow) and vertebrate (blue) hosts. Metabolic rewiring across stages reflects *T. cruzi’s* adaptability to dynamic host environments. *In culture, Epi and MTrypo utilize glucose primarily when available. **Despite glucose being available in host cells, it has been demonstrated that amastigotes (Ama) are unable to take up glucose (18). This simplified representation emphasizes the dominant carbon sources; other metabolites may contribute under specific conditions.

*T. cruzi* is transmitted in nature by blood-sucking triatomine insects. During their blood meal on an infected vertebrate host, the triatomines become infected with the parasite forms present in the bloodstream, the trypomastigotes (BTrypo) (17). These BTrypo, adapted to the glucose-rich vertebrate blood, prioritize glycolysis to meet their energetic demands (18–21). The BTrypo differentiate into replicative epimastigotes (Epi) in the insect’s midgut, which migrate to the hindgut. Here, nutrient scarcity, pH, and other environmental cues trigger the differentiation into non-replicative metacyclic trypomastigotes (MTrypo) (1–3, 22, 23). In addition to glucose, MTrypo is able to metabolize fatty acids and amino acids, such as proline, histidine, and glutamine, to sustain survival in the insect’s nutrient-poor environment (24–30). Transmission occurs when the triatomine, during a blood meal on a new vertebrate, defecates and releases together with the feces infective MTrypo on the skin or mucous membranes. Upon entering the host, MTrypo invades cells. Intracellularly, they differentiate into amastigotes (Ama), which exploit the host-cell cytoplasm to scavenge fatty acids, amino acids, and other metabolites, fueling their replication (27, 31–33). Finally, Ama differentiates into BTrypo, passing through an intermediate stage called intracellular epimastigote (Epi-like) (34). BTrypo bursts from host cells to disseminate via the bloodstream, completing the cycle (35, 36). This stage-specific metabolic reprogramming—from glycolysis to lipid and amino acid catabolism—highlights *T. cruzi*’s capacity to dynamically rewire its bioenergetic networks in response to hosts and their constraints (Fig. 2). Such adaptations ensure survival across disparate environments, underscoring the parasite’s evolutionary success as a pathogen.

### The glycosome

Glycosomes are specialized peroxisome-derived organelles in trypanosomatids that have compartmentalized glycolytic enzymes while retaining typical peroxisomal functions (37, 38). Found in Kinetoplastea and Diplonemea but absent in Euglenida, they likely evolved in the shared Euglenozoa ancestor (7, 39). In the 1980s, glycosomes were linked to other pathways, such as purine salvage, pyrimidine biosynthesis, and the pentose-phosphate pathway (PPP) (10, 37, 40), with proteomic studies later confirming these and additional enzymes (12, 13, 41). Notably, trypanosomatid glycolytic enzymes such as hexokinase (HK) and phosphofructokinase are largely unregulated (reviewed in reference 9), while cytosolic pyruvate kinase shows unique regulatory mechanisms (42). Like peroxisomes, glycosomes are permeable to small solutes (300–400 Da) (43), allowing the exchange of glucose (Glc) and most glycolytic and PPP intermediates with the cytosol. However, larger molecules like ATP, ADP, AMP, NAD^+^, and NADH are retained, creating distinct intraglycosomal pools (43). This separation necessitates intraglycosomal redox and ATP/ADP/AMP balance for the glycolytic pathway to proceed, which plays a role in mitigating risks associated with the pathway’s dangerous "turbo design” (44). The differing ATP/ADP and NAD^+^/NADH ratios between the glycosomes and the cytosol likely contribute to regulating carbon and energy metabolism in trypanosomatids (11, 43, 45).

### The mitochondrion

Unlike most eukaryotes, which have hundreds of mitochondria per cell, trypanosomatids possess a single mitochondrion that branches beneath the subpellicular microtubules, occupying approximately 13% of the cell volume (reviewed in reference 16). The mitochondrial DNA (kDNA) is localized in a specific region of the organelle near the basal body in a unique structure called the kinetoplast. The kDNA comprises interlocked circular DNA molecules (minicircles and maxicircles), forming a single network with species-specific sizes (14, 15). Located in the mitochondrial matrix, the kDNA is oriented perpendicular to the flagellum axis (reviewed in reference 16).

In trypanosomatids, including *T. cruzi*, mitochondria exhibit unique energy transduction mechanisms. Unlike typical eukaryotes, their mitochondria lack respiratory inhibition by rotenone, suggesting that complex I (NADH:ubiquinone oxidoreductase) is non-functional. Although all subunits of the electron transport system (ETS) are present, complex I appears incapable of proton pumping due to the absence of four crucial membrane subunits encoded by mitochondrial DNA (reviewed in reference 46). NADH oxidation is unaffected by antimycin, cyanide, or rotenone, pointing to an alternative NADH-oxidative system (47). Most of the rotenone-insensitive NADH oxidation in *T. cruzi* has been attributed to a mitochondrial NADH-fumarate reductase (mFR) (48, 49), which would reoxidize NADH to NAD^+^, transferring the electrons from NADH to fumarate and producing succinate. Succinate can then be reoxidized by complex II, feeding electrons into the ETS at the Q-junction. Another potential mechanism involves a rotenone-insensitive alternative NADH:quinone oxidoreductase (NDH2), though this has not been demonstrated in *T. cruzi*. In *T. brucei*, NDH2 sustains the mitochondrial inner membrane potential (∆Ψ_mt_), modulates sensitivity to ETS inhibitors, and regulates acetate biosynthesis (50, 51). Thus, while complex I’s role in *T. cruzi* remains unclear, electrons from complex I or NDH2 could still support oxidative phosphorylation (OxPhos) at the Q-junction. Notably, *T. brucei* possesses a fungal-like alternative oxidase (TAO) as its sole terminal oxidase in bloodstream forms (reviewed in reference 45). The absence of TAO homologs and enzymatic activity in *T. cruzi* and *Leishmania* spp. implies that these parasites lack the TAO-dependent glycosomal NADH oxidation system.

An important and unique function of TriTryps mitochondria is maintaining the redox balance within the glycosome, through the glycerol-phosphate shuttle coupled to a glycosomal NADH-fumarate reductase (gFR) (12, 43). Unlike other eukaryotes, where this shuttle operates in the cytosol, in TriTryps, it occurs within the glycosomes (52). Electrons from glycolytic intermediates are transferred to the Q-junction via the FAD-dependent mitochondrial glycerol-3-phosphate dehydrogenase (mG3PDH) (53), regenerating dihydroxyacetone phosphate (DHAP) in the glycosome and restoring intra-glycosomal NAD^+^ through glycosomal glycerol-3-phosphate dehydrogenase (gG3PDH). While glycerol-3-phosphate has been shown to stimulate respiration in *T. cruzi* Epi mitochondrial fractions (47), direct evidence for the shuttle’s functionality in *T. cruzi* remains limited.

The ability of *T. cruzi* mitochondria to generate endogenous reactive oxygen species (ROS) has not been thoroughly studied. However, the ETS has been identified as a major ROS source, primarily at complexes II and III, due to electron leakage during OxPhos (54). These ROS are not merely metabolic byproducts but also act as signaling molecules, influencing parasite differentiation, proliferation, and virulence (55–58). For example, ROS modulation is critical for life cycle transitions, such as from Epi to MTrypo (58), and ROS exposure enhances amastigogenesis from trypomastigotes (59). In addition, studies also suggest differential ROS production between Epi and BTrypo, with CII, CIII (54, 60), proline dehydrogenase (ProDH), and Δ1-pyrroline-5-carboxylate dehydrogenase (P5CDH) identified as key sites for mitochondrial ROS modulation in *T. cruzi* (61).

## CARBOHYDRATE METABOLISM

*T. cruzi* does not store sugars such as glycogen, amylopectin, or mannans (as in *Leishmania* spp.) (62–64). Although mammalian blood is nutrient-rich, the parasite’s presence there is transient. In the environments where it replicates, nutrients are scarce and inconsistently available. In the mammalian host-cell cytoplasm, the parasite competes for resources with the metabolic machinery and systems that sequester them into organelles. As a consequence, most metabolites are present at low concentrations (65) when compared with the *K*_*M*_ values for most of the parasite nutrient transporters characterized so far (66). In the insect midgut, *T. cruzi* faces rapid nutrient absorption by insect gut epithelial cells after blood meals (67–69). Consequently, in environments where Glc is often limited (70), *T. cruzi* relies on diverse carbon and energy sources, including other carbohydrates, to sustain homeostasis and replication.

Carbohydrate availability varies across host environments. In the 1970s, Zeledon and Lehmann identified fructose (Fru), mannose (Man), galactose (Gal), glucose (Glc), glucosamine (GlcN), and xylose (Xyl) as strong stimulators of respiration in Epi (71–73). Disaccharides like maltose, cellobiose, and melibiose slightly stimulated respiration, while others, such as 2-deoxy-D-glucose, trehalose, lactose, glycogen, starch, ascorbic acid, and glucuronate were ineffective (73). Although Epi preferentially metabolizes Glc in culture (19, 20, 71, 72), in the triatomine gut—where Epi and MTrypo naturally reside—carbohydrates are scarce. Nutrient absorption in the insect midgut occurs rapidly after a blood meal, forcing the parasite to compete for other resources (74). Additionally, insect-stage parasites are indirectly affected by the ecological stressors faced by the vector, such as variations in temperature and starvation (1–4, 75). Due to the scarcity of carbohydrates, it is believed that Epi and MTrypo rely on a broad variety of metabolites, such as amino acids and fatty acids from the triatomine intestinal lumen, as main carbon sources (2–4, 74). In the BTrypo, *T. cruzi* uptakes and uses Glc, which is abundant (~5 mM) in the bloodstream (19, 20, 76). However, hexose carrier (*Tc*HT) protein expression is drastically reduced in mammalian host stages, eliminating Glc transport in amastigotes (Ama) (18). Consequently, Ama likely cannot utilize other carbohydrates like Gal, Man, Fru, or GlcN (77, 78), relying instead on amino acids and fatty acids scavenged from the host cell.

### Glucose

Glc is transported into the cell via facilitated diffusion by *Tc*HT (78). Like other low-molecular mass metabolites, Glc freely diffuses between the glycosome and the cytosol, where the first six glycolytic enzymes convert it into 1,3-bisphosphoglycerate (1,3BPG) (7, 8, 10, 12, 40, 43, 79–86). In the cytosol, 1,3BPG is metabolized to phosphoenolpyruvate (PEP), which can either be converted to Pyr by a PK generating ATP (87) or return to the glycosome. There, PEP is carboxylated to oxaloacetate (OA) by phosphoenolpyruvate carboxykinase, producing intraglycosomal ATP (88, 89). OA is then reduced to malate by the glycosomal malate dehydrogenase (MDHg), reoxidizing NADH produced by glyceraldehyde-3-phosphate dehydrogenase (G3PDH) (90). Additionally, NADH can be reoxidized by the glycosomal NADH-dependent fumarate reductase, yielding succinate, which is excreted or transported into the mitochondria (48, 91). Since glycosomes are impermeable to adenylates and NADH, intraglycosomal redox (NAD^+^/NADH) and energy (ATP/ADP) balance must be maintained to sustain glycolysis (11, 44). Pyr serves as a metabolic branch point: it can enter mitochondria, where it is converted into acetyl-CoA, supplying carbons and redox power to the tricarboxylic acid cycle (TCAc) for the ATP-generating mitochondrial OxPhos system, or be transaminated to l-alanine (Ala), a major excretion product of *T. cruzi* (92, 93). Glycolytic intermediates like malate can also exit the glycosome. In the cytosol, malate is converted to Pyr by the cytosolic malic enzyme (cME) (94, 95) or transported to the mitochondria, where mitochondrial malic enzyme (mME) generates Pyr for further oxidation in the TCAc or transamination to Ala (95).

### Glucosamine

GlcN serves as a precursor for glycocalyx biosynthesis in *T. cruzi*. Its ability to inhibit Glc uptake indicates that GlcN is probably taken up by TcHT (77). Zeledon observed a significant increase in respiration with extracellular GlcN (73), though the degradative pathway linking this carbohydrate to glycolysis remains unclear. GlcN can be converted into GlcN-6P through HK and subsequently isomerized to fructose-6P (Fru-6P) via a putative glucosamine-6-phosphate isomerase. In this way, GlcN can proceed through the glycolytic pathway, thereby providing potential anaplerotic substrates. Despite this, almost no study has investigated the incorporation and use of these molecules in energy transduction pathways.

### Galactose

Gal is a key component of glycoconjugates in *T. cruzi* and is transported into the cell by a non-characterized system distinct from *Tc*HT (77, 78, 96). Zeledon noted a significant increase in respiration with extracellular Gal, though the metabolic pathway linking Gal to glycolysis (Leloir pathway and/or Isselbacher pathway) was not detailed (73). *T. cruzi* lacks the second enzyme of the Leloir pathway (galactose-1-phosphate uridylyltransferase), so Gal metabolism occurs via the Isselbacher pathway. This pathway operates in reverse to synthesize UDP-galactose from glucose-6P (Glc-6P), which is crucial for protein galactosylation (96). Notably, *T. cruzi* Epi can grow in a low-Glc medium supplemented with Gal, indicating its use as a primary carbon source (97). Once inside the cell, Gal is phosphorylated by galactokinase (GALK) to form Gal-1P (98), which is then converted to UDP-Gal by UDP-sugar pyrophosphorylase (USP) (99). UDP-Gal is epimerized to UDP-Glc by UDP-glucose-4-epimerase (GALE) (100). Through USP’s reverse activity, UDP-Glc is converted to Glc-1P, which is then transformed into Glc-6P by phosphoglucomutase (PGM) (101, 102). Glc-6P can subsequently enter glycolysis, as described earlier.

### Mannose and fructose

Like glucosamines and Gal, Man is a key component of *T. cruzi*’s glycocalyx and is transported into the cell by *Tc*HT (77, 78). Zeledon observed a significant increase in respiration with extracellular Man, though the degradative pathway linking Man to glycolysis remains unclear (73). For Man to enter glycolysis, it must first be phosphorylated. Urbina and Crespo demonstrated that Man is phosphorylated by an HK isolated and partially purified from *T. cruzi* (86). Mannose-6P is then converted to Fru-6P by phosphomannose isomerase, allowing it to enter glycolysis (12, 103). Like GlcN, Fru seems to be transported by *Tc*HT (77, 78). Fructose-specific HK activity has been detected in Epi cell-free extracts and glycosomal-enriched fractions (86), though not in recombinant HK (81), suggesting that a separate enzyme is responsible for this activity. Once phosphorylated to Fru-6P, Fru carbons can enter glycolysis, the TCAc, and stimulate respiration as noted by Zeledon (73).

### Xylose

Xyl is a monosaccharide found in glycoconjugates of the TriTryp (104). In *T. cruzi*, d-Xyl is linked to the phosphoserine/phosphothreonine-linked carbohydrate chain of the glycoprotein gp72 (104), which is associated with flagellar attachment. Xyl significantly stimulates respiration in *T. cruzi*, indicating it is transported and metabolized for ATP production (72). However, the transport system and catabolic pathways for Xyl in *T. cruzi* remain poorly understood. In other organisms, Xyl is metabolized to glycolytic intermediates (e.g., glyceraldehyde-3-phosphate) or enters the pentose phosphate pathway via conversion to xylulose and xylulose-5P (reviewed in reference 105). Yang and Bar-Peled proposed that *T. cruzi* converts Xyl into UDP-Xyl using a “sloppy” pyrophosphorylase (PPase), which forms UDP-sugars from diverse sugar-1-phosphates and UTP (99). UDP-Xyl may also be synthesized from other UDP-sugars, such as UDP-Glc and UDP-Gal (99). If these steps are reversible, as in human cells (106, 107), UDP-Xyl could be converted to UDP-Glc, which can then enter glycolysis via Fru-6P, similar to Gal metabolism (97).

## AMINO ACID METABOLISM

Although Epi incubated in Glc and amino acid-rich media preferentially consume Glc, they switch to amino acids and fatty acids (FA) as their energy and carbon sources when Glc is depleted (108–112). The abundance of these metabolites in the triatomine digestive tract or the excreta is poorly known, but *T. cruzi* Epi likely encounters amino acid-containing environments for two reasons: (i) during blood feeding, triatomines exhibit high proteolytic activity, efficiently degrading albumin and hemoglobin (the most abundant proteins in whole blood) to release free amino acids that the parasite can utilize (74); and (ii) feeding triatomines release high quantities of urea via Malpighian tubules, with amino acids leakage into the intestinal lumen (113, 114). While amino acid leakage is minimal during diuresis (<2% of the total present in hemolymph), under non-diuretic conditions, it can reach up to 90% of hemolymph concentration (114).

*T. cruzi* acquires amino acids through proteolytic activities, transport systems, or biosynthesis pathways. Amino acid transport systems for l-proline (Pro) (24), l-aspartate (Asp) (115), l-arginine (Arg) (116), l-glutamate (Glu) (117), l-glutamine (27), l-histidine (His) (28), l-leucine (Leu), l-isoleucine (Ile), l-valine (Val) (118), l-serine (Ser), l-threonine (Thr), and glycine (Gly) (66) among others, have been characterized in recent years (reviewed in reference 119). Except for His, transport occurs via active processes, dependent on the plasma membrane H^+^ gradient (reviewed in reference 119). This section reviews the current understanding of amino acid utilization for bioenergetic purposes in *T. cruzi*.

### Glutamate

Glu plays critical roles in *T. cruzi,* including osmoregulation, cell volume control (120, 121), and differentiation (122). It can be acquired from the environment through a saturable active transport system (117) or synthesized from metabolic sources and intermediates like Pro, His, Gln, acetate, and the TCAc intermediate alpha-ketoglutarate (α-KG) (123). Glu is reversibly converted to α-KG by glutamate dehydrogenases (GDH), releasing NH_4_^+^ and contributing to energy metabolism. Unlike metazoans, and similar to bacteria, fungi, and plants, *T. cruzi* Epi possess two GDHs (124, 125), one NADP^+^-linked, likely involved in biosynthesis (126), and one NAD^+^-linked, which increases its expression during the late exponential proliferation phase, when amino acids are consumed and NH_3_ is produced, suggesting a catabolic role (21, 86, 125, 127, 128). These GDHs, present in mitochondria and cytosol, may also reoxidize glycolytic NADH through Ala production and excretion (86, 128). Another way in which the NH_2_ group can be reversibly transferred from Glu to form α-KG is the transamination to α-keto acids by alanine aminotransferase or tyrosine aminotransferase (TAT), producing the corresponding amino acid (for example, Ala from Pyr) and α-KG. α-KG can then enter the TCAc, converted to malate, and subsequently to Pyr via malic enzymes, reentering the TCAc as acetyl-CoA. Glu (with Cys and Gly) also participates in the biosynthesis of glutathione, which is used in the biosynthesis of trypanothione, a key component of the redox defense mechanisms in trypanosomatids (129).

### Proline

Pro plays a vital role in *T. cruzi*’s biological processes. In Epi, it serves as a primary energy source in the absence of Glc (130). The parasite can acquire Pro through its transport via two active transport systems (24). Pro consumption is crucial for cell differentiation, invasion (26, 31, 61, 122, 131), resistance to oxidative imbalance (132, 133), and nutritional stress (134). Pro degradation occurs through two enzymatic reactions and one non-enzymatic reaction, which occur in the mitochondria. Pro is first oxidized by FAD-dependent ProDH, producing pyrroline-5-carboxylate (P5C) (133). Then, P5C is non-enzymatically converted into glutamate-γ-semialdehyde (GSA), which is then enzymatically converted to Glu by a P5C dehydrogenase (P5CDH) (135). Importantly, *T. cruzi* Pro can also be biosynthesized in the cytosol through two enzymatic and one non-enzymatic step. Firstly, Glu is converted into GSA by a P5C synthase (P5CS: γGK; GPR) (L. Marchese, R. W. Achjian, and A. M. Silber, unpublished results), which, after a non-enzymatic conversion into P5C, can be reduced by a P5C reductase (P5CR) to form Pro (136).

*T. cruzi* Epi can oxidize Pro with Pyr production from ^14^C-labeled Pro being observed (123). Beyond supplying Pyr for mitochondrial oxidation, the two enzymatic steps that convert Pro into Glu generate reduced equivalents to the ETS. ProDH reduces FAD to FADH_2_ (133) while P5CDH reduces NAD^+^ to NADH (135). Paes et al. demonstrated that FADH_2_ from ProDH directly feeds the ETS (133). The spontaneous interconversion between P5C and GSA does not involve a redox reaction, thus not contributing to the ATP production (118). Together, the two-step enzymatic oxidation of Pro to Glu generates FADH_2_ and NADH, fueling OxPhos. Additionally, Marchese et al. showed that P5CR (which catalyzes the reductive formation of Pro from P5C) is cytosolic and NADPH-dependent (136). Since ProDH is mitochondrial and delivers electrons into the ETS via FADH_2_, it was proposed that this cycle may translocate redox power to mitochondria under metabolic stress, supporting the ETS (136). These mechanisms explain Pro’s efficient use for ATP production in the absence of Glc, as demonstrated for Epi and MTrypo (25, 123, 133, 135).

### Histidine

His is an amino acid with well-documented antioxidant and anti-inflammatory properties across various organisms. This amino acid, along with cysteine, contributes to the formation of ovothiol A, a non-enzymatic scavenger of H_2_O_2_ (137). Despite its known biological roles and the presence of catabolic enzymes encoded in the *T. cruzi* genome, its function in these parasites remains poorly understood. Notably, trypanosomatids cannot biosynthesize His, making it an essential nutrient acquired exclusively from the extracellular environment (28). Research has demonstrated that His can be oxidized to CO₂, supply electrons to the respiratory chain, and promote ATP synthesis via oxidative phosphorylation (28). The His degradation pathway involves its conversion into Glu through four enzymatic steps: (i) histidine ammonium lyase removes the α-amino group, producing urocanate and ammonium (29, 138); (ii) urocanate hydratase converts urocanate into 4-imidazolone-5-propionate (IPA) (139); (iii) imidazolone propionase (IP) hydrolyzes IPA’s amide bond, yielding N-formimino-l-glutamate; and (iv) formimine glutamase directly hydrolyzes the formimine group to produce Glu and formamide (140).

### Glutamine, aspartate, and asparagine

*T. cruzi* acquires Gln through two main mechanisms: uptake from the culture medium through a highly specific, low-affinity transport system (27, 141) and biosynthesis via glutamine synthetase (GS) (141). GS catalyzes the ATP-dependent conversion of Glu and NH₄^+^ into Gln (27, 141). Beyond Gln synthesis, GS is proposed to play a critical role in NH₄^+^ detoxification, as *T. cruzi* lacks the urea cycle, a primary NH₄^+^ elimination pathway in other organisms (142). GS also contributes to the parasite’s ability to invade host cells. During the transient phase in the parasitophorous vacuole, *T. cruzi* relies on maintaining a low pH, escaping from the vacuole into the cytoplasm, and establishing infection. Inhibition of GS disrupts this process, increasing the vacuole’s pH, preventing escape, and impairing host-cell infection (141). In addition, Gln serves as a nitrogen donor in multiple pathways: (i) the hexosamine pathway, via Gln fructose-6-phosphate aminotransferase (27); (ii) the pyrimidine biosynthesis pathway, as a substrate of carbamoyl-phosphate synthase (143, 144); (iii) the GMP synthesis pathway, through GMP synthase (145); and (iv) the production of Asn through glutamine amidotransferase (GlnAT) (146). Importantly, all these reactions regenerate Glu, which can be oxidized to produce ATP. Moreover, it has been demonstrated that carbon derived from Gln participates in the biosynthesis of sterols in Ama (32).

Asp can be transported from the extracellular medium by a transporter system likely shared with Glu (115) or synthesized by the reversible amination of oxaloacetate, a TCAc intermediate, catalyzed by Asp aminotransferases (147). Asp triggers O_2_ consumption in *T. cruzi* mitochondria (73) and has been detected as labeled in cells incubated with ^14^C-l-Pro, indicating a unidirectional carbon flux from Pro to Asp (123). Some enzymes of the Asp metabolism have been identified in *T. cruzi*: (i) Asp aminotransferase in Epi, which can interconvert Glu and Asp using oxaloacetate and α-KG as carbon backbones (148, 149); (ii) Asp carbamoyltransferase, which synthesizes carbamoyl–aspartate from carbamoyl–phosphate and Asp (150); and (iii) adenylosuccinate synthase, which condensates Asp with IMP, forming adenylosuccinate. This intermediate, in turn, can be converted into fumarate via adenylosuccinate lyase, thus contributing to the TCAc (151).

Recent findings from our laboratory showed that all life stages of *T. cruzi* can uptake Asn ( L. Marchese and A. M. Silber, unpublished data). Asn can also be synthesized from Asp by an Asn synthetase using NH4^+^ as a nitrogen source, in a reaction that is ATP-dependent (152). The catabolism of Asn happens through its deamidation into Asp, which can enter the TCAc after being converted into oxaloacetate through transamination (147). This pathway allows Asn to stimulate mitochondrial O_2_ consumption (73).

### Serine, threonine, glycine, and alanine

In most environments colonized by *T. cruzi*, Ser, Thr, Ala, and Gly are nearly ubiquitous nutrients (113, 153). Despite their abundance, their role in the energy metabolism of *T. cruzi* remains poorly understood. While most organisms synthesize Ser *de novo* from 3-phosphoglycerate (3PG) (154), *T. cruzi* lacks the final two steps of this pathway. Instead, it likely relies on uptake and/or biosynthesis via Ser hydroxymethyltransferase, which produces Ser from glycine (Gly) and 5,10-CH_2_-THF in an irreversible reaction (155). Regarding Thr, while plants, bacteria, and fungi synthesize it *de novo* from Asp (156, 157), *T. cruzi* lacks the first three steps of this pathway. It has been proposed that *T. cruzi*, like *T. brucei*, might synthesize Thr from homoserine and acyl-homoserine lactones, bypassing the homoserine kinase step (158). Due to these biosynthetic deficiencies, *T. cruzi* depends on the extracellular uptake of Ser and Thr (66). Recent studies show that Ser and Thr are important carbon and energy sources for *T. cruzi* during starvation, directly contributing to ATP production (66). Serine/threonine dehydratase converts Ser and Thr into Pyr and 2-oxobutanoate, respectively, while Gly does not contribute to the energy metabolism (66). Additionally, Thr can be catabolized by the mitochondrial enzyme l-threonine-3-dehydrogenase in tandem with 2-amino-3-ketobutyrate ligase, producing Gly and acetyl-CoA (159). In *T. brucei*, this pathway connects Thr with intramitochondrial ATP production via the acetate:succinate CoA transferase/succinyl-CoA synthetase (ASCT/SCS) cycle and supports acetate production, a key step for cytosolic lipid biosynthesis (160–163). As previously mentioned, Gly also participates in the biosynthesis of glutathione and thus, of trypanothione (129).

Ala, along with succinate, is one of the end products of Glc metabolism by Epi. It is produced through the reversible amination of Pyr (92, 93). Under conditions of excess NH_4_^+^, Ala can be synthesized by alanine dehydrogenase or through the combined action of an NAD^+^-linked GDH and aminotransferases that utilize Pyr as a substrate. This process may also facilitate the reoxidation of glycolytically produced NADH, even in the presence of oxygen. Depending on the substrates and product concentrations, Ala can be converted back into Pyr by the same enzymes. Notably, intracellular and secreted pools of Ala are compartmentalized and produced separately (164). Despite being an excreted metabolic end product, Ala can be transported back into the parasite and metabolized. It has been shown to stimulate O_2_ consumption and ATP production under starvation conditions, highlighting its role as an energy source (165).

### Branched-chain amino acids

*T. cruzi* cannot synthesize branched-chain amino acids (BCAA) (Leu, Ile, and Val) *de novo*. Therefore, they must be acquired from the extracellular environment via a specific, active, and saturable transport (118). BCAA has been shown to inhibit Pro metabolism, thereby impairing metacyclogenesis (166–168). In *T. cruzi*, it has been demonstrated that Leu and Ile are oxidizable carbon sources, stimulating O_2_ consumption in starved Epi (73). Although *T. cruzi* lacks genes or canonical branched-chain amino acid aminotransferases, it performs BCAA deamination by transamination using tyrosine and aspartate aminotransferases in Epi and Ama, respectively (169). The predicted catabolic pathways for BCAAs share several enzymes, and their products can feed into the TCAc: Leu yields acetoacetate and acetyl-CoA (ketogenic), Val generates succinyl-CoA (glucogenic), and Ile produces propionyl-CoA and acetyl-CoA (glucogenic and ketogenic, respectively). O_2_ uptake of *T. cruzi* is depressed when Leu is the sole substrate but recovers when Glc is added. Notably, Leu does not interfere with Glc metabolism, indicating different oxidation routes (170, 171). CO_2_ production from ^14^C1-Leu and increased O_2_ consumption in Epi support this connection, though Glc supplementation reduces Leu oxidation by half. In addition to its involvement in ATP synthesis through OxPhos, Leu-derived acetoacetate and acetate can yield acetyl-CoA and acetoacetyl-CoA, substrates for the ASCT/SCS cycle, supporting ATP production by mitochondrial substrate-level phosphorylation and lipid biosynthesis in the cytosol. Notably, *T. cruzi* synthesizes small amounts of sterol from Leu, but it uses far more effectively acetate for sterol biosynthesis when compared to *Leishmania* spp. (172).

### Cysteine and methionine

Cysteine (Cys) plays a critical role in protein structure, stability, and catalytic function (173). Although *T. cruzi* can synthesize Cys *de novo*, it is also actively transported into Epi through a specific transport system (174). While Cys can contribute to energy metabolism through its conversion into Pyr (175), its primary role is likely linked to redox metabolism (176, 177), serving as the main source of –SH groups for sulfur-containing molecules like glutathione (Glu, Cys, and Gly adduct), trypanothione (glutathione and spermidine), and ovothiol (His and Cys adduct) which are essential for resistance to oxidative imbalance (137, 178).

Methionine’s (Met) function in *T. cruzi* has not been as well studied as Cys. There is no evidence of an active Met biosynthetic pathway in *T. cruzi*, so it is presumed that this amino acid needs to be taken up from the extracellular medium (179), although Met transport has still not been characterized. In addition to its participation in protein synthesis, Met is involved in redox balance (179) and methylation processes by the formation of S-adenosyl methionine (SAM), the main methyl donor in most organisms (180). *T. cruzi* presents a putative gene annotated in its genome that may encode an S-adenosyl methionine synthetase.

Cys biosynthesis in *T. cruzi* can occur through the transsulfuration and the *de novo* pathways. The transsulfuration pathway involves the formation of cystathionine from Ser and homocysteine (HCys) by cystathionine β-synthase (181, 182). Then, cystathionine is cleaved into Cys and 2-aminobut-2-enoic acid by cystathionine γ-lyase (181, 182). HCys is presumed to be derived from Met via SAM and S-adenosyl homocysteine intermediates, though only S-adenosyl homocysteine hydrolase, the final enzyme in this pathway, has been characterized in *T. cruzi* (183–185). The *de novo* biosynthesis involves the activation of Ser by acetyl-CoA to form O-acetylserine (OAS), catalyzed by serine O-acetyltransferase (181). OAS reacts with free SH_2_ to form Cys and acetate, mediated by cysteine synthase (181, 186). Both pathways likely work together to meet Cys demands. However, the presence of a Cys transport highlights the importance of maintaining intracellular Cys homeostasis (174), especially given the low sulfur-containing amino acid in the Reduviidae gut (30). Cys degradation produces Pyr, which can enter energy metabolism (187, 188). Mercaptopyruvate sulfurtransferase converts mercaptopyruvate into Pyr, releasing H₂S. Although *T. cruzi* lacks cysteine aminotransferase, aspartate aminotransferase can use Cys as a substrate to produce mercaptopyruvate (147, 189). Overall, Cys serves not only as an energy source but also as a key regulator of redox balance in *T. cruzi* Epi (174).

### Arginine: arginine-phosphate system

l-Arg is crucial for the energetic metabolism of *T. cruzi* (190). Since *T. cruzi* lacks the urea cycle (191), Arg must be acquired through the two high-affinity, specific transport systems (116, 192, 193). Inside Epi, Arg is primarily metabolized into phosphoarginine (ArgP) (116, 194), an important energy reserve. This system is analogous to the creatine–phosphocreatine phosphagen system in vertebrates, resulting in a high-energy phosphate bond exchange (116, 195). ArgP reversibly transfers its phosphate to ADP, generating ATP via arginine kinase (AK) (194). The proliferation rate of Epi correlates with AK activity, which increases during exponential proliferation and peaks in the stationary phase, suggesting that AK regulates energy reserves under starvation stress (196). Over-expression of *T. cruzi* AK enhances parasite proliferation and survival under nutritional and pH stress (197). Additionally, AK expression increases in Epi exposed to hydrogen peroxide, indicating its role in oxidative stress responses (198). Remarkably, overexpression of the Arg transporter TcAAP3 raises intracellular Arg levels, shifting the AK reaction equilibrium to increase ArgP, with a concomitant reduction of ATP (199).

### Aromatic amino acids

Aromatic amino-acid catabolism is active in trypanosomatids, though it is less complete than in mammals, rarely degrading them fully to CO_2_ and water (200). In *T. cruzi,* two key enzymes are involved: TAT and an aromatic l-2-hydroxy acid dehydrogenase (AHADH) (92, 200–204). TAT uses Tyr, Phe, or Trp as -NH_2_ donors to α-keto acid, yielding the corresponding aromatic 2-oxoacids as subproducts. AHADH then reduces these oxoacids into aromatic hydroxy acids, producing the corresponding aromatic lactates. This pathway is proposed to serve as a mechanism for cytosolic NADH re-oxidation, linking aromatic amino acids to redox balance.

### Lysine

*T. cruzi* likely acquires Lys through more than one transport system (193), though only one Lys transporter gene has been identified and characterized so far (205). Beyond its obvious participation as a constituent of proteins, the biological relevance of Lys in *T. cruzi* remains unclear. *In silico* analysis, preliminary metabolomics studies, and searches for enzymatic activities related to Lys biosynthesis or degradation have not yet revealed a specific metabolic role for this amino acid in the parasite.

### d-Amino acids: alanine, serine, and proline

d-Amino acids play diverse structural and physiological roles in various processes, including peptidoglycan biosynthesis, biofilm modulation, and neuromodulation (206–210). In *T. cruzi*, d-amino acids can be acquired through transport or via racemases. Evidence exists for d-Pro uptake (132) and proline racemase activity (211). Additionally, the genome encodes for a putative alanine racemase. Unpublished findings from our group suggest that *T. cruzi* expresses a functional alanine racemase, which, due to its absence in mammals, is promising as a drug target. While the physiological role of d-amino acids in *T. cruzi* remains poorly understood, they may contribute to defense mechanisms against proteolysis through the production of d-amino acid-containing peptides (211, 212). The conversion of d-enantiomers of Ala, Ser, and Pro enables the metabolic connection of these amino acids with the canonical degradation pathways mentioned earlier (Pyr-forming transaminases, serine dehydratase, and Pro degradative pathway), expanding the parasite’s potential carbon sources available within host environments. Other d-amino acids such as d-Asp, d-Thr, d-Val, and d-Ser were identified in the Epi soluble extract, suggesting that these amino acids may be transported or intracellularly racemized (212). Despite the potential of the enzymes producing them as drug targets, their role and metabolism in *T. cruzi* biology have been scarcely investigated.

## FATTY ACID METABOLISM

*T. cruzi* Epi efficiently incorporates free FA (213, 214). FA oxidation (FAO) is critical for the parasite’s bioenergetics, with NADP^+^-dependent β-oxidation of palmitoyl-CoA occurring in both mitochondrial and glycosomal fractions (215). Proteomic studies confirmed the presence of all necessary enzymes in glycosomes (12). Palmitic, linoleic, linolenic, and stearic acids are taken up by *T. cruzi,* metabolized into CO_2_, and incorporated into neutral lipids and phospholipids (109, 112). Palmitic acid shows the highest incorporation rate. Notably, FA oxidation to CO_2_ is more pronounced in MTrypo than in Epi, while FA incorporation into lipids is greater in Epi (109, 112). This observation is consistent with the fact that Epi replicates, which requires an active biosynthesis of membranes.

*T. cruzi* Epi stores significant amounts of neutral lipids, primarily cholesterol and cholesterol esters, within reservosomes sourced from the extracellular environment (216). Lipid-starved parasites rely on these stored lipids for immediate metabolism, likely to support membrane production and proliferation (217). The presence of an acyl-CoA-cholesterol acyltransferase-related enzyme highlights the role of exogenous cholesterol in Epi lipid metabolism. Additionally, lipid droplets form in the posterior region of Epi, with variations in metabolic stages, maturation levels, or lipid composition observed in them (218).

Regarding other parasite stages, little information is available on their ability to metabolize lipids. A recent study showed that Ama proliferation relies on the host pyruvate dehydrogenase activity regulation and FAO. This suggests a possible mechanism of *T. cruzi* to preferentially thrive in host environments, favoring FAO over glycolysis (33).

A study by our group revealed that *T. cruzi* Epi undergoes a metabolic shift from Glc consumption to FAO under Glc scarcity. This shift is regulated by the downregulation of acetyl-CoA carboxylase to suppress FA synthesis and the upregulation of carnitine palmitoyltransferase 1 (CPT1) to promote FAO (108). This metabolic flexibility sustains ATP production, enhancing survival under metabolic stress. FAO fuels oxidative phosphorylation by supplying electrons to the ETS and acetyl-CoA to the TCAc, maintaining mitochondrial activity (108). Inhibition of CPT1 with etomoxir, which blocks FA translocation into the mitochondria, significantly reduces O_2_ consumption, mitochondrial function, and ATP levels, underscoring *T. cruzi’s* reliance on FAO for bioenergetic stability (108). Free FA also induces cell differentiation to infective forms in *T. cruzi* (213). FAO is essential for surviving prolonged starvation and plays a key role in differentiation, as blocking FA metabolism impairs metacyclogenesis. Glc suppresses FAO, indicating a regulatory interplay between carbohydrate and lipid metabolism, where Glc is preferred when available, but FAO becomes vital during Glc depletion (108). These findings highlight the importance of FA as an alternative energy source supporting *T. cruzi*’s survival, mitochondrial function, and differentiation.

Trypanosomes rely on an alternative pathway for most FA synthesis, mediated by elongases (219). Pagura et al. generated knockout lineages of the Fatty Acids Elongases 1-3 (ELO 1-3), confirming their functionality and distinct roles. The ELO1 knock-out lineage showed increased metabolites, reduced ΔΨ_mt_, and elevated mROS, suggesting impaired FAO and dysregulated energy metabolism. In contrast, ELO2 and ELO3 are involved in shaping the global lipidome of *T. cruzi* Epi (110).

## OTHER ENERGY SOURCES

### Acetate and pyruvate

Acetate, a common product of Glc degradation in *T. cruzi* Epi, is oxidized via TCAc, involving mitochondrial ETS, as evidenced by the inhibitory effect of antimycin A (220). Labeled acetate is incorporated into Glu, Ala, Gly, and TCAc intermediates, succinic acid, and malic acid, with their carboxyl carbon released as CO_2_. Acetate carbons were found in the skeletal carbon of Asp and Glu (220). Over 50% of the ^14^C coming from labeled acetate is incorporated into Ala and Glu, as well as intermediates of glycolysis (220). Different from Epi, adding acetate to the BTrypo notably increased the respiratory rate (221). Acetate is oxidized by the BTrypo until CO_2_, in an antimycin A-inhibitable way (221). Additionally, acetate is efficiently used by *T. cruzi* for sterol biosynthesis (172).

Few studies have investigated the use of Pyr as an exogenous carbon source. When combined with Pro, Pyr increases the O_2_ consumption rate in Epi (222) and moderately stimulates O_2_ consumption when it is the sole exogenous carbon source (123). Although de Boiso and Stoppani showed that Pyr is transported in Epi (220). However, the transport system has not yet been characterized.

### Glycerol

Since Zeledon reported that glycerol stimulates *T. cruzi* respiration (73), its metabolism in the parasite has been largely unexplored. However, studies in *T. brucei* highlight glycerol’s role in energy metabolism and gluconeogenesis (223–227). Like glycolysis, glycerol metabolism is compartmentalized in glycosomes (12). Glycerol is initially transported into the cells via an unknown mechanism and phosphorylated by a glycosomal glycerol kinase, forming glycerol-3-phosphate (G3P) (228). G3P can follow two pathways: (i) it is transported to the cytosol and used by mitochondrial glycerol-3-phosphate dehydrogenase, donating electrons at the Q-junction; or (ii) it is converted to DHAP by glycosomal glycerol-3-phosphate dehydrogenase, entering glycolysis to produce pyruvate. Glycerol can be a valuable carbon source during scarcity, and investigating its metabolism in *T. cruzi* could reveal key mechanisms of metabolic flexibility.

## CONCLUDING REMARKS

*T. cruzi* exhibits unique metabolic features that raise intriguing evolutionary questions and present opportunities for discovering novel therapeutic interventions. The parasite demonstrates remarkable flexibility in utilizing carbohydrates, amino acids, and lipids, adapting to environmental challenges throughout its life cycle. Among its distinctive characteristics, *T. cruzi* (as other trypanosomatids) compartmentalizes pathways typically cytosolic in other organisms into a peroxisome-derived organelle called the glycosome and possesses a single mitochondrion per cell, which relies not only on OxPhos but also on SubPhos for ATP production. Regarding the ETS and NADH reoxidation, complex I appears unable to extrude protons to contribute to ΔΨ_m_. Instead, a fumarate reductase seems responsible for NADH reoxidation by converting fumarate to succinate, which is then reoxidized in complex II, generating FADH_2_.

Beyond Glc, other carbohydrates, such as Man, Gal, GlcN, and Xyl, can feed glycolysis at various points, ensuring Pyr and ATP production. Most amino acids also contribute to OxPhos by generating NADH and/or FADH_2_, which donate electrons to the ETS through the pathways described above. For instance, His, Pro, and Gln produce Glu, which is deaminated to α-KG, while Asn generates Asp and OA, both feeding the TCAc. The BCAAs and Thr can power ATP synthesis by producing acetyl-CoA in a Pyr-independent way, while Ala, Cys, and Ser directly generate Pyr. Arg plays a unique role in cellular energy management. Although not directly involved in ATP biosynthesis, it can be reversibly phosphorylated from ATP, forming ArgP and ADP, creating an additional reservoir of high-energy phosphate bonds that can be reconverted into ATP as needed. Finally, fatty acids can be acquired from the environment or mobilized from lipid droplets for mitochondrial ATP production, likely through the production of acetyl-CoA by β-oxidation. Most of the data collected from the literature were determined mainly in Epi and to a lesser extent in BTrypo, which are easily obtained from cultures. Unfortunately, knowledge of Ama metabolism remains scarce. A concerted effort to understand the biology and metabolism of Ama and Epi-like forms could provide valuable insights for the rational development of stage-specific drugs targeting stages of clinical relevance. A compilation of relevant data can be found in Table 1. In summary, *T. cruzi*’s metabolism enables it to thrive in diverse environments throughout its life cycle. Its ability to efficiently switch between carbohydrates, amino acids, and lipids as energy sources highlights its metabolic versatility. These adaptations not only provide insights into the evolutionary aspects of *T. cruzi* metabolism but also open new avenues for discovering new therapeutic targets.

**TABLE 1:** Uptake and utilization of energy and carbon sources by *Trypanosoma cruzi^a^*

Molecule	Uptake	Energy source	Carbon source	Reference
Glucose	Epi, BTrypo, MTrypo	Epi, MTrypo, BTrypo	Epi, MTrypo, BTrypo	(18–21, 71, 72, 77)
Glucosamine	Epi	Epi	ND	(73, 77, 78)
Mannose	Epi	Epi	ND	(73, 77, 78)
Fructose	Epi	Epi	ND	(72, 77, 78)
Galactose	Epi	Epi	Epi, BTrypo	(96, 97, 229, 230)
Xylose	ID	Epi	ND	(72)
l-Proline	Epi	Epi, MTrypo	Epi-Like, Ama	(24, 25, 31, 61, 123, 132, 133, 136)
d-Proline	Epi	ND	ND	(26, 131, 211, 212)
l-Glutamate	Epi	Epi	Epi	(117, 124–128, 148)
l-Glutamine	Epi, MTrypo, BTrypo, Epi-Like, Ama	ND	Ama	(27, 32, 141)
l-Aspartate	Epi	Epi	ND	(115, 148)
l-Asparagine	ID Epi	ND	ND	(117)
l-Histidine	Epi	Epi	ND	(28, 29)
l-Alanine	Epi	Epi	ND	(92, 93, 128, 164, 165)
d-Alanine	ID Epi	ND	ND	(165)
l-Serine	Epi, MTrypo, BTrypo, Epi-Like, Ama	Epi	Epi	(66, 231)
D-serine	ID Epi	ND	ND	(66)
Glycine	Epi	Epi	ND	(66, 155, 232)
l-Threonine	Epi	Epi	Epi	(66, 159)
l-Cysteine	Epi	ND	Epi	(174, 181, 182)
l-Methionine	ND	ND	ND	ND
l-Leucine	Epi	Epi	Epi	(118, 172)
l-Isoleucine	Epi	ND	ND	(118)
l-Valine	Epi	ND	ND	(118)
l-Arginine	Epi	Epi	Epi	(116, 192, 193)
Phenylalanine	ND	ND	Epi	(201)
Tryptophan	ND	ND	Epi	(201)
Tyrosine	ND	ND	Epi	(201, 202)
l-Lysine	Epi	ND	ND	(193, 205)
Acetate	Epi, BTrypo	Epi, BTrypo	Epi, BTrypo	(172, 220, 221)
Pyruvate	Epi	Epi	Epi	(220)
Glycerol	ID Epi	Epi	ND	(73, 228)
Fatty acids	Epi	Epi, Ama	Epi, MTrypo, Ama, BTrypo	(33, 108–110, 213, 229, 230)

^*a*^ The table indicates in which life cycle stages there is evidence to support the processes of transport, energy metabolism, and use as a carbon source. Epi, epimastigote; MTrypo, metacyclic trypomastigote; BTrypo, bloodstream trypomastigote; Ama, amastigote; Epi-like, intracellular epimastigote. ND, no data; ID, indirect data.
